# The machine learning algorithm based on decision tree optimization for pattern recognition in track and field sports

**DOI:** 10.1371/journal.pone.0317414

**Published:** 2025-02-13

**Authors:** Guomei Cui, Chuanjun Wang

**Affiliations:** College of Physical Education, Shandong Sport University, Rizhao, China; University of Lagos Faculty of Engineering, NIGERIA

## Abstract

This study aims to solve the problems of insufficient accuracy and low efficiency of the existing methods in sprint pattern recognition to optimize the training and competition strategies of athletes. Firstly, the data collected in this study come from high-precision sensors and computer simulation, involving key biomechanical parameters in sprint, such as step frequency, stride length and acceleration. The dataset covers multiple tests of multiple athletes, ensuring the diversity of samples. Secondly, an optimized machine learning algorithm based on decision tree is adopted. It combines the advantages of Random Forest (RF) and Gradient Boosting Tree (GBT), and improves the accuracy and efficiency of the model in sprint pattern recognition by adaptively adjusting the hyperparameter and tree structure. Specifically, by introducing adaptive feature selection and ensemble learning methods, the decision tree algorithm effectively improves the recognition ability of the model for different athletes and sports states, thus reducing the over-fitting phenomenon and improving the generalization ability. In the process of model training, cross-validation and grid search optimization methods are adopted to ensure the reasonable selection of super parameters. Moreover, the superiority of the model is verified by comparing with the commonly used algorithms such as Support Vector Machine (SVM) and Convolutional Neural Network (CNN). The accuracy rate on the test set is 94.9%, which is higher than that of SVM (87.0%) and CNN (92.0%). In addition, the optimized decision tree algorithm performs well in computational efficiency. However, the training data of this model comes from the simulation environment, which may deviate from the real game data. Future research can verify the generalization ability of the model through more actual data.

## Introduction

As the core event in track and field events, sprint has extremely high requirements for athletes’ explosive power, pace frequency, stride length and starting reaction [1]. Accurate identification and analysis of these key movements is very important for improving training methods, improving sports performance and preventing sports injuries [2]. However, the traditional manual evaluation and observation methods are subjective and limited, and it is difficult to meet the refined needs of modern sprint training [3]. At present, the research of sprint technical action recognition mainly focuses on the application of sensor technology, signal processing and machine learning algorithm [4]. Machine learning algorithm realizes the recognition and classification of athletes’ movements by automatically learning the key features in a large number of sprint data [5]. Especially, machine learning algorithms based on decision tree optimization, such as Random Forest (RF) and Gradient Boosting Tree (GBT), have shown great application potential in the field of sprint technical action recognition because of their excellent classification performance, robustness and strong adaptability to data noise. RF enhances the stability of the model by integrating multiple decision trees and reduces the risk of over-fitting, while gradient lifting tree finely adjusts the prediction results of the model by gradually optimizing. These optimization methods can help the model to better capture the key technical actions in sprint, such as starting reaction, acceleration stage and sprint stage.

With the progress of machine learning technology and the development of data acquisition technology, data-driven motion pattern recognition has shown broad application prospects in sprint technical action analysis [6]. In this study, large-scale sports data from several sprinters are collected, including accelerometer (X, Y, Z axis), heart rate, step frequency and other sensor data. These data are collected in real time by high-precision sensors, and the quality and consistency of the data are ensured by preprocessing steps (such as denoising and standardization). Machine learning algorithm can identify and classify different technical actions by automatically learning and extracting these features, and provide scientific data support and decision-making basis for coaches and athletes [7]. Machine learning algorithms based on decision tree optimization, such as Random Forest (RF) and Gradient Boosting Decision Tree (GBDT), have great application potential in the field of sprint technical motion recognition because of their excellent classification performance and good robustness [8].

The purpose of this study is to use the machine learning algorithm based on decision tree optimization to realize accurate identification and classification of various technical motions by analyzing and processing large-scale sprint data. The significance of the study lies in improving the scientificity and accuracy of athletes’ training, helping coaches to better understand and evaluate athletes’ technical movements, thus optimizing training plans, improving training effects and reducing the risk of sports injuries. This not only contributes to the technical improvement and competitive performance improvement of sprint, but also provides new methods and ideas for the research in sports science and sports medicine.

The structure of this study is mainly divided into the following sections. First, it summarizes the current research status of sprint technical motion recognition. Second, it expounds the application of machine learning algorithm based on decision tree optimization in sprint technical motion recognition, including the principle and optimization process of RF and GBDT. Third, it describes the source, research object and data preprocessing method of dataset to ensure the quality and consistency of data. The experimental results and performance evaluation are presented to verify the effectiveness and practicability of the proposed algorithm in sprint technical action recognition. Finally, the shortcomings of the study are analyzed, the future research direction and improvement measures are put forward, and the potential significance and value of this study in practical application are discussed.

## Literature review

Sprint is the core event in track and field, and its high intensity and high technical requirements make it very important to accurately identify athletes’ action patterns [9]. Sprinters need to accurately control the movement rhythm and power output in training and competition, especially in the starting, acceleration and sprint stages [10]. The traditional technical evaluation method mainly relies on the observation and experience of coaches, which has the defects of strong subjectivity and poor reproducibility [11]. In contrast, the sensor-based data acquisition method can provide objective, real-time and accurate sports data support, and has become an important tool for sprint technical analysis. However, these traditional sensor methods also have limitations in application, for example, when the sensor is single or the data is insufficient, it is difficult to fully reflect the athletes’ sports characteristics at different stages, and the recognition effect of complex sports patterns is limited. By analyzing large-scale and high-dimensional motion data, machine learning method can automatically extract complex motion features from the data and effectively classify different motion patterns. This enables machine learning to overcome the limitations of traditional methods in dealing with big data, identifying complex patterns and improving real-time. For example, Prasanth et al. (2021) collected the lower limb movement data of sprinters through inertial measurement unit, and studied the movement characteristics at different running stages. The research showed that sensor technology could not only provide high-precision data support, but also realize real-time monitoring, which is helpful to identify and improve sprint technical movements [12].

In recent years, machine learning algorithm has been widely used in sprint technical action recognition [13]. Algorithms such as support vector machine, random forest and deep neural network are used in the research of motion pattern recognition [14, 15]. These algorithms can extract features from a large number of motion data and realize the classification of complex motion patterns. For example, Al-Faris et al. (2020) used RF algorithm to analyze the gait data of sprinters, and successfully identified the action patterns in different stages, demonstrating the effectiveness of machine learning technology in sprint movement pattern recognition [16]. Deep learning technology, especially convolutional neural network (CNN), has demonstrated strong feature extraction ability in the field of motion pattern recognition. Su et al. (2020) used CNN to process the data from wearable devices, classified the action patterns of sprinters, and achieved remarkable results. This method not only significantly improves the accuracy, but also can deal with complex multi-dimensional data [17]. Under the background that machine learning algorithm is applied to sprint technical action recognition, machine learning technology in other fields also shows significant potential. For example, in recent years, the application of machine learning in human-machine interface control design has made remarkable progress. The research shows that machine learning algorithm can optimize the design of human-machine interface and improve the control accuracy and response speed of the system. For example, Zhang et al. (2022) combined the Max-Min Ant System and genetic algorithm with machine learning, optimized the layout through cognitive model in the design, and achieved efficient system control performance [18]. The advantage of these algorithms is that they can automatically adjust system parameters, realize efficient layout design and optimization, and thus enhance the overall experience of human-computer interaction.

Although a lot of progress has been made in the field of sprint technical motion recognition, there are still some shortcomings. Many studies only focus on a single stage of sports, lacking a comprehensive analysis of the whole sprint process [19, 20]. At the same time, the existing machine learning model may face problems such as high computational complexity and poor real-time performance when dealing with large-scale data [21]. In addition, the noise and inconsistency of sensor data will also affect the accuracy and robustness of the model [22]. Over-fitting is a common problem in machine learning model, especially in the case of small training data or too many features. Over-fitting will lead to the model performing well on the training set, but its prediction ability on unknown data is poor, which affects its generalization ability. Therefore, the regularization and cross-validation strategy of the model is particularly important to ensure the generalization ability and stability of the model. Another potential problem is the generalization ability of the model in different types of sprint data or between different athletes. The model may be accurate in the performance of some athletes, but it may not perform well in the data of other athletes. Therefore, data enhancement and transfer learning are needed to improve the adaptability and generalization ability of the model. In addition to sensor technology, machine vision has also made a breakthrough in the field of human motion recognition, especially in the extraction and application of temporal and spatial motion features. Motion recognition based on machine vision often uses spatio-temporal video volume model to describe the motion process, and uses spatio-temporal interest point method to extract features for action classification. For example, Arunnehru et al. (2022) proposed a spatio-temporal motion feature descriptor based on the difference intensity distance group pattern, which was used to effectively identify human actions in videos. This method greatly improved the accuracy of motion recognition by capturing local motion and being invariant to size and shape changes [23]. In the classification stage, the research also used classification methods such as SVM and RF, and achieved excellent recognition results. Although this research mainly focused on the processing of visual data, its thinking also had important enlightenment for sprinters’ action recognition. Especially when it comes to complex motion pattern recognition, the combination of machine learning and video analysis is helpful to improve the accuracy and real-time performance of sprint motion recognition system.

To make up for the above shortcomings, this study intends to use the machine learning algorithm based on decision tree optimization to accurately identify the action patterns of sprinters in key stages such as starting, accelerating and sprinting by analyzing and processing large-scale sprint data. The innovation of this study lies in the dynamic analysis of the whole sprint by combining sensor technology and deep learning algorithm, aiming at providing coaches and athletes with more scientific technical evaluation and training suggestions to optimize the training effect and reduce the risk of sports injury.

## Materials and methods

### Data source, research object, and data preprocessing

The sprint dataset generated based on computer simulation is used. The dataset contains several key parameters (time stamp, speed, acceleration, step frequency, stride length and heart rate), which are of great significance in sprint. Each data point represents the instantaneous motion state in a sprint, covering the whole process from the start to the finish line. Using computer simulation tools to generate sprint data based on physical model, the simulation tool generates data according to the classical biomechanical model and the specific conditions of sprint (such as different starting postures, acceleration stages, maximum speed maintenance and so on).

The following factors are considered in the simulation process: speed curve: It describes the change of speed with time in the sprint process, from standstill to maximum speed, and then to deceleration at the end. Step frequency and stride length: It simulates the change of step frequency and stride length of athletes in different stages. Acceleration: It records the acceleration changes of athletes in the starting and accelerating stages. Heart rate simulation: Based on the known human physiological model, it simulates the change of heart rate during sprint.

The dataset structure is as follows: Timestamp: the time point of each record. Speed: the speed of movement in meters per second. Acceleration: acceleration in meters per second. StepFrequency: the step frequency in steps per second. StepLength: the step in meters. HeartRate: the heart rate in seconds per minute (analog data). The data is collected at high frequency, and the sampling rate is set at 100Hz to ensure that every detail change in the sprint process is captured. 100 sprints are simulated, and the length of each sprint is 10 seconds. The total data volume is 4.8MB.

Each simulated sprint process contains about thousands of data points. The dataset includes several simulation scenarios, covering different starting conditions, athletes’ physical conditions and so on.

Data preprocessing is one of the key steps before sprint pattern recognition. This study’s data preprocessing includes data cleaning and standardization to ensure data quality and consistency, thereby enhancing the accuracy and efficiency of ML models. The objective of data cleaning is to remove or correct noise, missing values, and outliers in the dataset. Specific steps include using mean imputation for missing values. For instance, if heart rate data is missing for a certain period, it is filled using the mean heart rate of the athlete from other periods. The process can be described in Eq (1).


$${HR}_{\mathrm{new}}=\frac{\sum_{i=1}^{n}\;{HR}_{i}}{n}$$ (1)

*HR*_*new*_ represents the new heart rate data; $\sum_{i=1}^{n}\;{HR}_{i}$ represents the value filled in by mean interpolation; *n* means the sum of all effective heart rate data.

The Z-score method is used to detect and process outliers, as shown in Eq (2).

$$Z=\frac{X-\mu }{\sigma }$$ (2)

*X* refers to the numerical value of a single data point; *σ* and *μ* represent the standard deviation and mean of the dataset.

Z-score can measure the degree of deviation between data points and the mean, and usually sets a threshold of 3. If the Z-score absolute value of a data point is greater than 3, it is considered an outlier and replaced with the mean of neighboring data points.

Low-pass filters are utilized to remove high-frequency noise. For example, high-frequency noise in heart rate and accelerometer data is smoothed using a moving average filter, as expressed in Eq (3):

$$y(t)=\frac{1}{N}\sum_{i=t-N+1}^{t}\;x(i)$$ (3)

*y*(*t*) represents the smoothed data point; *N* indicates the window size of the moving average filter; $\sum_{i=t-N+1}^{t}\;x(i)$ refers to the sum of the data points within the window and is used to calculate the average.

The variations before and after data cleaning are exhibited in Table 1.

**Table 1:** Variations before and after data cleaning (partial).

Data type	Before cleaning	After cleaning
**Heart rate**	85, 90, NaN, 88, 300	85, 90, 89, 88, 89
**Accelerometer data (X-axis)**	1.2, 1.3, -3.4, 1.1, 1.4	1.2, 1.3, 1.2, 1.1, 1.4

The purpose of data standardization is to convert data of different dimensions into the same dimension and eliminate the scale difference between data. This study adopts the Z-score standardized method [24, 25]. In the standardization process, the mean and standard deviation of each motion parameter (such as heart rate and accelerometer data) are calculated as follows:

$$\begin{array}{c} \mu =\frac{\sum_{i=1}^{n}X_{i}}{n} \end{array}$$ (4)

$$\sigma =\sqrt{\frac{\sum_{i=1}^{n}{\left(X_{i}-\mu \right)}^{2}}{n}}$$ (5)

The changes in data before and after standardization are outlined in Table 2.

**Table 2:** The changes in data before and after standardization (partial).

Data type	Before standardization (sample)	After standardization (sample)
**Heart rate**	85, 90, 89, 88, 89	-1.41, 0.35, 0.00, -0.35, 0.00
**Accelerometer data (X-axis)**	1.2, 1.3, 1.2, 1.1, 1.4	-0.45, 0.45, -0.45, -1.34, 1.34

The ratio of training set to test set in the dataset is 8:2.

### The ML algorithm based on decision tree optimization

To improve the accuracy and efficiency of sprint pattern recognition, this study adopts a machine learning algorithm based on decision tree optimization, including RF and GBT. These algorithms improve the model performance by adaptively adjusting parameters and structures [26]. The reason behind this choice is that these algorithms perform well in dealing with high-dimensional data and complex feature spaces. Multi-dimensional characteristics such as heart rate and acceleration involved in sprint need to be identified and distinguished by accurate models. RF improves the robustness and accuracy of the model by integrating the prediction results of multiple decision trees. GBT can further optimize the model performance by gradually reducing the prediction error. This study combines the advantages of these two algorithms, and makes in-depth algorithm optimization and parameter adjustment.

RF is an ensemble learning method using decision trees, which improves the accuracy and robustness of the model by constructing multiple decision trees and combining their output results [27, 28]. The main characteristics of RF are diversity and ensemble learning. The mathematical representation of RF reads:

$$h(X)=\frac{1}{N}\sum_{i=1}^{N}\;h_{i}(X)$$ (6)

*h*(*X*) represents the *i*th decision tree’s prediction result, and *N* indicates the number of decision trees.

**Fig 1:**
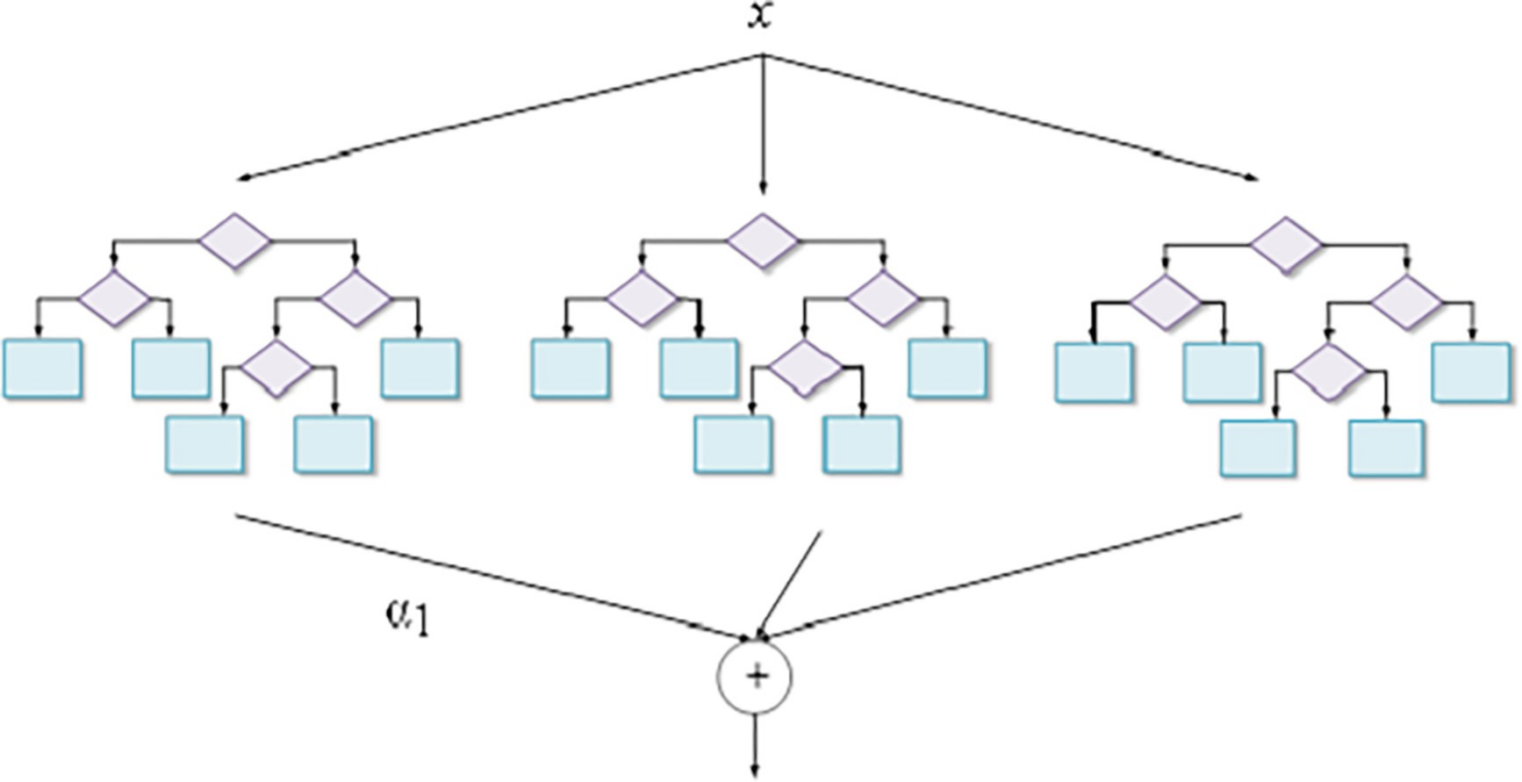
The structure of GBDT.

GBDT is an ML algorithm that optimizes the objective function by building decision trees step by step [29, 30]. The structure of GBDT is presented in Fig 1:

Unlike RF, GBDT builds each tree in a serialized manner, and each tree is constructed based on the residual of the previous tree, gradually reducing the prediction error. The main features of GBDT are residual fitting and weighted summation [31]. The mathematical representation of GBDT can be written as Eq (7):

$$F_{m}(x)=F_{m-1}(x)+\gamma \cdot h_{m}(x)$$ (7)

*F*_*m*_(*x*) denotes the prediction result of the *m*th round; *h*_*m*_(*x*) represents the *m*th tree, and *γ* is the learning rate.

Optimization and parameter adjustment are carried out to fully leverage the advantages of RF and GBDT. This study optimizes the model’s hyperparameters through grid search and cross-validation methods [32]. The hyperparameters include the depth of the decision tree (*d*), the minimum sample sizes to split nodes (*m*), and the learning rate (*γ*), as listed in Table 3:

**Table 3:** The range of hyperparameters.

Model	RF	GBDT
**Number of decision trees**	100–1000	100–1000
**The maximum depth of each tree**	10–100	3–10
**Minimum sample size/learning rate for split nodes**	2–10	0.01–0.1

The performance calculation of the hyperparameter combination reads:

$$\underset{n,d,m,\gamma }{min}\frac{1}{K}\sum_{k=1}^{K}\;L(y^{\left(k\right)},{\hat{y}}^{\left(k\right)}(n,d,m,\gamma ))$$ (8)

*K* represents the fold of cross-validation; *L* refers to the loss function. This study chooses the cross-entropy function.

In this study, an integrated learning model based on decision tree optimization is used, including RF and GBT. In the design of the model, the input features include heart rate, accelerometer data (X, Y, Z axis) and so on. The framework of the model is as follows: random forest: the input layer contains multi-dimensional motion features, and each feature is processed by different decision trees, and finally the prediction results are output through voting mechanism. The main hyperparameter settings of random forest include: the number of decision trees: 500; Maximum depth of each tree: 50; Minimum number of samples of split nodes: 5. GBT: each decision tree is built in turn, and each tree is built based on the residual of the previous tree. The parameters are set as follows: the number of decision trees: 300; Maximum depth of each tree: 5; Learning rate: 0.05. The activation function of the model is ReLU, and the loss function is Cross-Entropy to optimize the classification performance of the model. In the process of super-parameter optimization, Grid Search and Cross-Validation are used to find the optimal parameter combination.

Combining the advantages of RF and GBDT, the model’s robustness and accuracy are improved by the weighted fusion method. The mathematical representation of the fusion model is as follows:

H(X)=α⋅RF(X)+(1−α)⋅GBDT(X) (9)

*α* denotes the weight of RF; *RF*(*X*) and *GBDT*(*X*) represent the predicted results of RF and GBDT, respectively.

The weighted fusion method combines the advantages of RF and Gradient Boosting Decision Tree (GBDT) to improve the accuracy and robustness of the model. In the process of weighted fusion, the model optimizes the weighting coefficient α of each sub-model. Specifically, the weighting coefficient is obtained by evaluating experiments with different α values on the verification set. The advantages and disadvantages of each sub-model are weighed in the fusion process, thus enhancing the performance of the overall model. It should be noted that the weighted fusion method can effectively balance the advantages and disadvantages of each sub-model and improve the accuracy and reliability of the overall model when dealing with different types of data.

To determine the optimal weight α, the following optimization process is carried out [33]:

1. Initial weight: The initial weight *α*_0_ = 0.5 is set.
2. Weight adjustment: Multiple attempts are made on the validation set to adjust the value of *α* in steps of 0.1.
3. Performance evaluation: The accuracy of the fusion model under each weight is calculated, and the weight with the highest accuracy is selected as the final *α*.

Using accuracy as the evaluation indicator, the weight optimization process is denoted in Table 4:

**Table 4:** The weight optimization process.

Process steps	Description	Weight (*α*)	Accuracy (%)
**Initialize weights**	Setting initial weight *α* = 0.5	0.5	94.2
**Weight adjustment (1)**	Adjusting weight *α* = 0.4	0.4	93.7
**Weight adjustment (2)**	Adjusting weight *α* = 0.6	0.6	95.8
**Weight adjustment (3)**	Adjusting weight *α* = 0.7	0.7	94.9

Through the above process, the optimal weight α = 0.6 is determined, which means that 60% of the weight in the fusion model comes from RF and 40% of the weight comes from GBDT.

Feature importance analysis is used to identify the features that have the greatest impact on model prediction [34]. In this study, decision tree algorithm is used to evaluate the contribution of each feature to the model. In the decision tree, each feature is used as a split node at different nodes of the tree, so that the contribution of each feature to the tree model can be calculated. Specifically, information gain and feature selection frequency are two commonly used indicators to measure the importance of features. Information Gain: Information gain is a measure of the purity improvement brought by a feature in the decision tree. Whenever a feature is used as a splitting node, the model will calculate the uncertainty or entropy that the feature can reduce, thus obtaining information gain. The characteristics of high information gain usually have a great influence on the prediction performance of the model. Feature Selection Frequency: The feature selection frequency indicates the number of times a feature is used as a split node in the decision tree. Frequently selected as a split node, it shows that it plays an important role in classification decision. Through the above analysis, the following key features are identified: heart rate, pace frequency, stride length and accelerometer data.

Features often do not act independently, but affect the final prediction results through interaction. In the performance prediction of sprint, there may be strong interaction among the characteristics such as step frequency, stride length and heart rate, which means that considering the influence of each characteristic alone may not fully reflect its role in the actual sports process. For example, the interaction between step frequency and stride: There is usually an inverse relationship between step frequency and stride, and increasing step frequency may lead to the shortening of stride, which directly affects the running efficiency and speed of athletes. Interaction between heart rate and pace frequency: In sprint, the athlete’s heart rate usually rises with the increase of exercise intensity. The interaction between heart rate and pace frequency may reflect the endurance and exercise intensity of athletes, which has an important impact on sprint performance.

In order to better understand and quantify the interaction between features, Shapley value method is further used in this study for detailed analysis. Shapley value is a tool from cooperative game theory, which is used to quantify the contribution of each feature to model prediction and capture the interaction between features. Shapley value provides a way to evaluate the contribution of each feature to the prediction results in all possible feature combinations based on fair distribution. For each feature, it considers the predicted changes brought by adding this feature into the model, and also considers the interaction between this feature and other features.

Super parametric optimization is a key step to improve the accuracy and performance of the model. In order to ensure the best performance of the decision tree optimization model in this study, a combination of Grid Search and Random Search is adopted to determine the optimal super parameter combination through cross-validation.

In this study, Convolutional Dual-Stage Attention-Based Long Short-Term Memory (CDLSTM) model is used to optimize the parameters of convolution layer and Long Short-Term Memory (LSTM) layer. Through grid search, experiments are carried out under different learning rates, the number of LSTM units and the size of convolution kernel to ensure that the model can learn the most effective features from time series. In addition, in order to further improve the accuracy and robustness of the model, the nonparametric optimization method of RF and GBDT model is adopted. The results are shown in Table 5:

**Table 5:** Super parameter optimization result.

Super parameter combination	Number of Trees	Max Depth	Min Samples Split	Learning Rate	Accuracy	Training Time, s	Other Metrics
**Combination 1**	100	10	5	0.1	92.50%	120	Precision: 0.90, Recall: 0.85, F1: 0.87
**Combination 2**	200	15	10	0.05	93.20%	180	Precision: 0.92, Recall: 0.88, F1: 0.90
**Combination 3**	300	20	15	0.05	94.10%	210	Precision: 0.93, Recall: 0.89, F1: 0.91
**Combination 4**	400	25	20	0.01	94.50%	250	Precision: 0.94, Recall: 0.90, F1: 0.92
**Combination 5**	500	30	25	0.01	95.00%	300	Precision: 0.95, Recall: 0.91, F1: 0.93
**Combination 6**	1000	50	30	0.001	94.80%	450	Precision: 0.94, Recall: 0.90, F1: 0.92

According to Table 5, Combination 5 (number of decision trees is 500, maximum tree depth is 30, minimum sample number is 25, and learning rate is 0.01) has the best performance in terms of precision, recall rate and F1 score, with a precision of 95.0%. The performance of other combinations is also good, but the training time will increase with the number and depth of trees. Therefore, it is necessary to balance the performance and computational efficiency of the model in practical application.

### Model training methods and experimental environment

Model training and verification is an important step to ensure that the model has high accuracy and stability in sprint pattern recognition. The cross-validation method is adopted for model training and multiple performance evaluation indicators are employed to evaluate the model’s performance.

Cross-validation is an effective method for evaluating model performance and avoiding overfitting. This study uses the K-fold cross-validation method for model validation [35]. The specific steps are as follows:

1. Data segmentation: The dataset is randomly divided into K equally sized subsets.
2. Training and validation: One subset is selected as the validation set, and the remaining K-1 subset is used as the training set. This is repeated K times, each time selecting a different subset as the validation set.
3. Average results: The results of K verifications are averaged to obtain the overall performance evaluation of the model.

K-fold cross-validation is an important method to evaluate the generalization ability of the model. K = 10 is selected for cross-validation. In order to verify the influence of K value on the model performance, experiments with different K values (such as K = 5, K = 10, K = 20) are carried out in this study. The results show that the selection of K value will affect the training time of the model and the stability of the verification results. Specifically, too small a value of K may lead to the instability of the verification results, while too large a value of K will increase the calculation burden. Therefore, K = 10 is finally chosen as the optimal discount. The specific expression is as follows:

$${CV}_{K}=\frac{1}{K}\sum_{i=1}^{K}\;{Perf}_{i}$$ (10)

*CV*_*K*_ means the average performance of K-fold cross-validation, and Perf_*i*_ represents the performance metric of the *i*th validation.

The advantage of cross-validation is that it can fully utilize the dataset for training and validation and reduce the randomness caused by different data partitions. Furthermore, it can better evaluate the model’s performance on unseen data.

To comprehensively evaluate the performance of the model in sprint pattern recognition, the following commonly used performance evaluation indicators are used, covering accuracy, precision, recall, and F1 score, as given in Eqs (11)–(14):

$$\mathrm{Accuracy}=\frac{TP+TN}{TP+TN+FP+FN}$$ (11)

$$\mathrm{Precision}=\frac{TP}{TP+FP}$$ (12)

$$Recall=\frac{TP}{TP+FN}$$ (13)

$$F1\;\mathrm{Score}=2\times \frac{Precision\times Recall}{Precision+Recall}$$ (14)

*TP* and *TN* represent true positive and true negative; *FP* and *FN* refer to false positive and false negative.

The value of Area Under the Curve (AUC) is also added as a performance evaluation index. AUC is the area under the Receiver Operating Characteristic Curve (ROC), reflecting the model’s overall classification performance. The closer the AUC value is to 1, the better the model performance.

The software and hardware environment of this experiment is detailed in Table 6:

**Table 6:** Experimental environment.

Specific level	Detailed information
**Hardware**	Desktop computers with Intel Core i7 processors, 16 Gigabytes (GB), Random Access Memory (RAM)
	Nvidia GeForce Ray Tracing Technology (RTX) 2080 Graphics Processing Unit (GPU)
**Software**	Python 3.8
	Scikit-learn 0.24.2
	TensorFlow 2.6.0
	Jupyter Notebook 6.4.5
	Pandas 1.3.3
	NumPy 1.21.2

In the application of machine learning model, there are many challenges, especially the influence of over-fitting, model generalization ability and training conditions on model performance. In order to ensure the validity of the model, the following points are considered in this study:

Over-fitting problem: In order to avoid model over-fitting, cross-validation (such as K-fold cross-validation) and regularization techniques (such as L2 regularization) are adopted. In addition, the pruning operation of decision tree also effectively prevents the model from over-fitting on the training set.

Model generalization ability: Early stop technology is used to prevent the model from being over-optimized on the training set, thus improving the generalization ability of the model. In addition, the model is verified on several different datasets to ensure that it can handle the test data under different conditions.

Influence of training conditions: Training conditions such as training set size, data distribution and noise will affect the performance of the model. In order to reduce the influence of data bias, Synthetic Minority Over-sampling Technique (SMOTE) technology is used to enhance the data, and several rounds of experiments are conducted to evaluate the stability of the model.

In addition, this study also uses CDLSTM model for training to further improve the time series learning ability of the model, especially when dealing with time series data. In addition, SMOTE with Deep Neural Networks (SMOTEDNN) algorithm is also introduced to balance data sets and solve the problem of data imbalance.

## Results and discussion

### Feature importance score

The results of feature importance score based on information gain and feature selection frequency are shown in Table 7:

**Table 7:** Feature importance score based on information gain and feature selection frequency.

Characteristic	Information gain score	Feature selection frequency	Comprehensive importance score
**Stride frequency**	0.32	0.27	0.295
**Step**	0.25	0.23	0.24
**Heart rate**	0.28	0.22	0.25
**Acceleration (x)**	0.15	0.18	0.165
**Acceleration (y)**	0.14	0.17	0.155
**Acceleration (z)**	0.13	0.15	0.14

Table 7 shows that the comprehensive importance score is obtained by combining the information gain score with the feature selection frequency. According to the table, it shows that the step frequency and heart rate have the greatest influence on the model, and these two characteristics are given priority.

Shapley value analysis results are shown in Table 8:

**Table 8:** Shapley value analysis results.

Characteristic	Shapley value (main effect)	Step frequency × stride interaction	Step frequency × heart rate interaction	Step × heart rate interaction	Total contribution
**Stride frequency**	0.35	0.12	0.15	0.08	0.7
**Step**	0.3	0.12	0.1	0.14	0.66
**Heart rate**	0.25	0.09	0.18	0.11	0.63
**Acceleration (x)**	0.1	0.03	0.05	0.02	0.2
**Acceleration (y)**	0.08	0.02	0.03	0.02	0.15
**Acceleration (z)**	0.07	0.01	0.02	0.01	0.11

In Table 8, Shapley value analysis shows the main effect (single action) of each feature and its interaction with other features. Step frequency, stride length and heart rate all contribute to the main effect and interaction, especially the interaction between step frequency and stride length and the interaction between step frequency and heart rate. The acceleration data has little influence on the model.

### Analysis of the results of the cross-validation of the model

**Fig 2:**
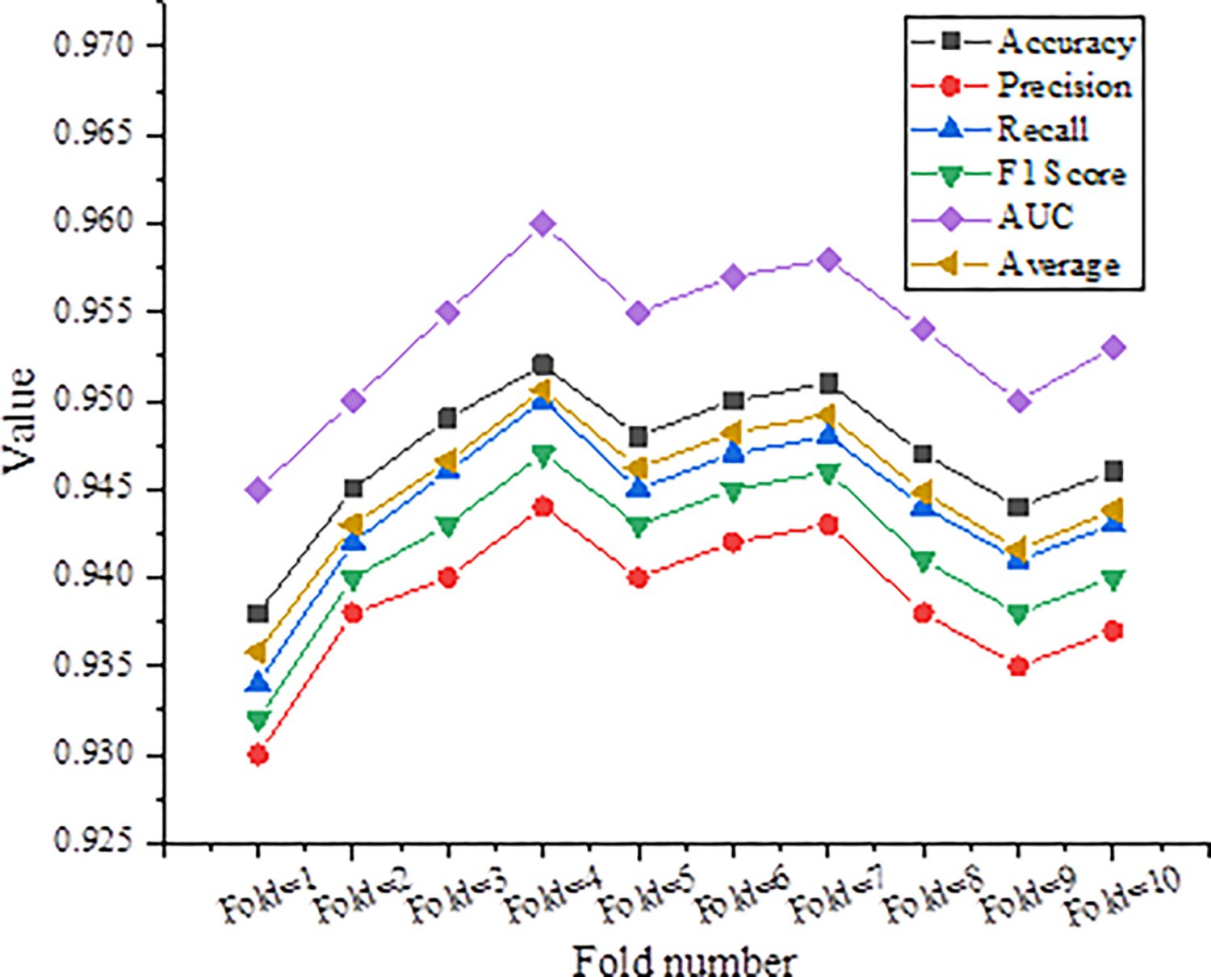
Results of cross-validation of the proposed model.

By dividing the dataset into 10 equally sized folds, cross-validation is performed. In each iteration, one fold is chosen as the validation set while the remaining 9 folds serve as the training set. This process is repeated 10 times, each time using a different fold as the validation set. The model’s performance in terms of accuracy, precision, recall, F1 score, and AUC values at various fold numbers is evaluated, as demonstrated in Fig 2.

In Fig 2, the machine learning algorithm based on decision tree optimization performs well in all indicators of 10-fold cross-validation. The accuracy fluctuates slightly between the folds, maintaining between 93.8% and 95.2%, with an average accuracy of 94.57%. The performance of precision rate and recall rate are also very close, ranging from 93.0% to 94.4% and 93.4% to 95.0% respectively. The F1 score ranges from 93.2% to 94.7%. AUC values are higher than 0.94 under different folding numbers, which shows that the model can maintain relatively stable and good classification performance under various decision thresholds, and can effectively distinguish positive and negative samples.

### Comparative analysis of model performance

**Fig 3:**
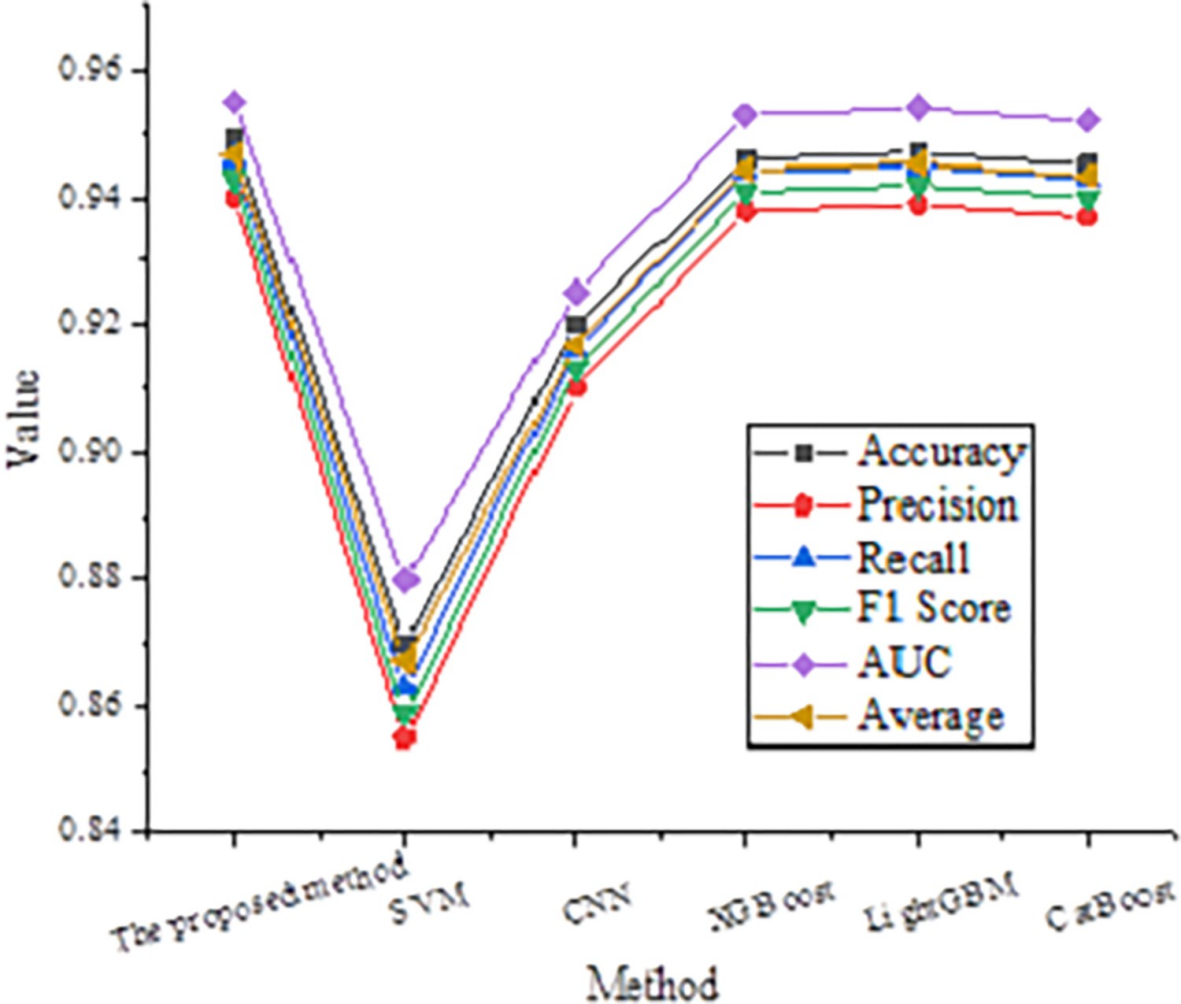
Performance comparison of different models.

The proposed algorithm’s performance comparison analysis with five other common ML algorithms is presented. The compared algorithms include SVM, Convolutional Neural Network (CNN), Extreme Gradient Boosting (XGBoost), Light Gradient Boosting Machine (LightGBM), and CatBoost. The comparison results are depicted in Fig 3.

In Fig 3, the machine learning algorithm based on decision tree optimization is superior to other algorithms in various performance indexes. The accuracy of this model is 94.9%, which is higher than SVM (87.0%), CNN (92.0%) and slightly higher than XGBoost (94.6%), LightGBM (94.7%) and CatBoost (94.5%). The accuracy, recall and F1 score are all higher than 93%, which is the best among all the comparison algorithms. In addition, the AUC value of this model is 0.955, which shows that the model has high robustness and accuracy under different classification thresholds.

### Comparative analysis of computational efficiency of models

**Fig 4:**
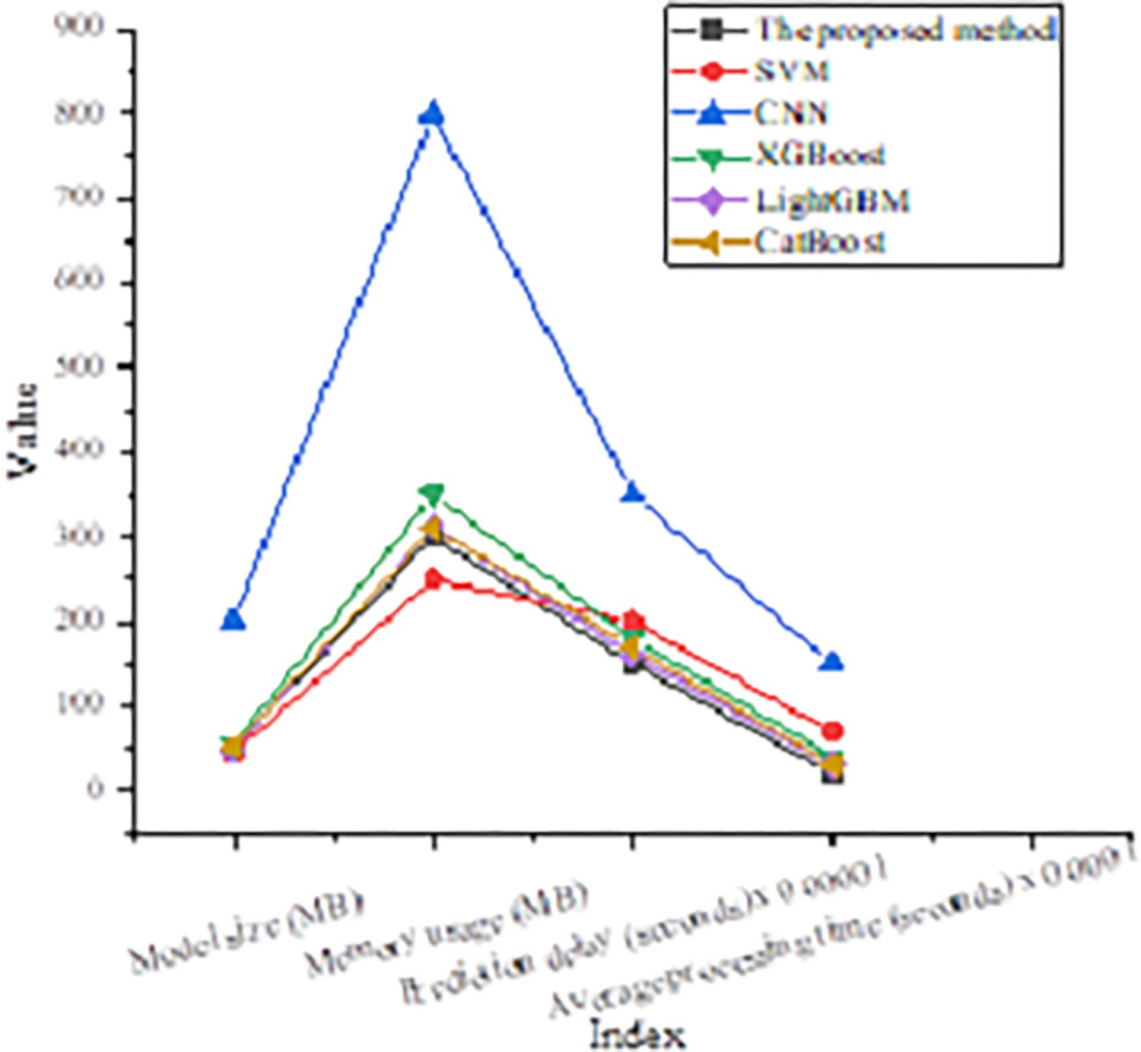
Comparison of the computational efficiency of different models.

A comparative analysis of computational efficiency is conducted between the proposed algorithm and the other five common ML algorithms. Apart from the average time taken to process each data record, four metrics are compared: training time, model size, memory usage, and prediction latency. The results are suggested in Fig 4:

As shown in Fig 4, the proposed model’s size is 50 Megabytes (MB), which is moderate, smaller than SVM (45MB) and LightGBM (48MB) in terms of lightweight processing, but larger than CNN (200MB). The memory usage during training and prediction for the proposed model is 300MB, better than CNN (800MB) but slightly higher than the other algorithms. The decision tree optimization-based ML algorithm excels in all computational efficiency metrics, particularly in training time and prediction latency, meeting the real-time and efficiency requirements of track and field sports pattern recognition.

## Discussion

The model design of the study skillfully combines the advantages of RF and GBT, and achieves a good balance in stability and accuracy. RF effectively reduces the risk of over-fitting of a single decision tree by integrating multiple decision trees, and improves the robustness of the model in sprint data. At the same time, the gradient lifting tree makes the model perform well in capturing the complex data patterns in the process of sprint through gradual optimization. This combination strategy makes the model not only have the stability of random forest, but also have the high accuracy of gradient lifting tree in dealing with sprint data. In the process of model optimization, adaptive parameter adjustment and structural optimization methods are adopted to make the model dynamically adapt to the characteristics of sprint data. This adaptive adjustment strategy not only significantly improves the prediction accuracy of the model, but also performs well in computational efficiency. Through many experiments and verification, the best parameter combination is determined, which makes the model realize the optimization of performance in the process of data training and prediction of sprint. The effectiveness of this optimization process has been fully verified in the comparison between model performance and computational efficiency. In this study, although the model performs well on simulated datasets, for larger datasets, especially those involving high dimensions or complexity, the computational efficiency and scalability of the model are still important issues to be considered. The computational efficiency of the model remains good in most cases, but with the expansion of the dataset, the time cost of training and prediction may increase significantly. Although this study improves the training efficiency of the model by optimizing the algorithm and parallel computing, the training time and memory consumption of the model also show an increasing trend with the increase of data volume. In the face of more complex high-dimensional datasets, although the model reduces the feature dimension through ensemble learning and feature selection, the complexity may still lead to an increase in computing time, especially without distributed computing. Therefore, for larger datasets, the model may encounter scalability problems, especially in memory consumption and computing speed. Future research can further explore how to improve the efficiency of the model in big data environment through distributed computing framework. In addition, for the deep learning model with high computational complexity, more incremental learning or online learning strategies may be needed to update the model step by step without retraining the whole dataset to better adapt to large-scale data processing.

## Conclusion

The innovation lies in the dynamic analysis of the whole sprint by combining sensor technology and deep learning algorithm, aiming at providing more scientific technical evaluation and training suggestions for coaches and athletes, thus optimizing the training effect and reducing the risk of sports injuries. Specifically, grid search and cross-validation methods are employed to optimize the model’s hyperparameters. Weighted fusion techniques are also used to enhance the model’s robustness and accuracy. Research results demonstrate that the ML algorithm based on decision tree optimization performs exceptionally well across all performance metrics. Compared to common ML algorithms such as SVM, CNN, XGBoost, LightGBM, and CatBoost, the proposed model shows remarkable advantages in accuracy, precision, recall, and F1 score. Moreover, the proposed model exhibits good computational efficiency, meeting the real-time and efficiency requirements of track and field sports pattern recognition in terms of training time and prediction latency. However, this study still has some limitations. Firstly, there is a need to expand the dataset’s diversity and scale to cover more types of track and field sports and a broader range of athlete samples. Secondly, the model’s robustness and generalization capabilities need further validation across different scenarios. In addition, the proposed model also performs well in calculation efficiency, and the training time and prediction delay can meet the requirements of real-time and high efficiency for sprint pattern recognition. However, there are still some shortcomings. Firstly, the diversity and scale of the dataset need to be further expanded to cover more types of sprints and more athletes’ samples. Secondly, the robustness and generalization ability of the model need to be further verified in different scenarios. Future research directions include further optimizing the methods of feature engineering and data preprocessing, and exploring more effective feature extraction and selection technologies. Meanwhile, more advanced machine learning and deep learning algorithms can be considered to further improve the performance and robustness of the model. In addition, further research on the biomechanical data of sprint can provide more high-dimensional and detailed motion data for the model, which may enhance the model’s ability to identify patterns. By digging deep into the individual differences of athletes, such as the biomechanical characteristics, physical condition and training history of athletes, people can tailor a more personalized training plan for each athlete and effectively optimize the training effect. Specifically, the combination of high-precision sensor data and more detailed individual data of athletes can further improve the model’s ability to predict athletes’ action patterns, performance and potential risks, and then provide more accurate technical support and optimization suggestions for scientific training of sprint events. The practical significance of this study is that by improving the algorithm of sprint pattern recognition, the ability to analyze and optimize athletes’ technical movements is improved, which lays a foundation for the future application in the field of athletes’ personalized training and sports performance optimization.

## Supporting information

S1 Dataset (ZIP)

## References

[1] Pu B, Li K, Li S, Zhu N. Automatic fetal ultrasound standard plane recognition based on deep learning and IIoT. IEEE Transactions on Industrial Informatics. 2021; 17(11): 7771–7780.

[2] Moin A, Zhou A, Rahimi A, Menon A, Benatti S, Alexandrov G, et al. A wearable biosensing system with in-sensor adaptive machine learning for hand gesture recognition. Nature Electronics. 2021; 4(1): 54–63.

[3] Zhang Z, Sun C. Structural damage identification via physics-guided machine learning: a methodology integrating pattern recognition with finite element model updating. Structural Health Monitoring. 2021; 20(4): 1675–1688.

[4] Muaaz M, Chelli A, Gerdes M W, & Pätzold M. Wi-Sense: A passive human activity recognition system using Wi-Fi and convolutional neural network and its integration in health information systems. Annals of Telecommunications. 2022; 77(3): 163–175.

[5] Verma K K, Singh B M, & Dixit A. A review of supervised and unsupervised machine learning techniques for suspicious behavior recognition in intelligent surveillance system. International Journal of Information Technology. 2022; 14(1): 397–410.

[6] Ferrari A, Micucci D, Mobilio M, Napoletano P. Deep learning and model personalization in sensor-based human activity recognition. Journal of Reliable Intelligent Environments. 2023; 9(1): 27–39.

[7] Khan M M, Hossain S, Mozumdar P, Akter S, & Ashique R H. A review on machine learning and deep learning for various antenna design applications. Heliyon. 2022; 8(4). doi: 10.1016/j.heliyon.2022.e09317 35520616 PMC9061263

[8] Bharadiya J P, Tzenios N T, & Reddy M. Forecasting of crop yield using remote sensing data, agrarian factors and machine learning approaches. Journal of Engineering Research and Reports. 2023; 24(12): 29–44.

[9] Gabbett T J. How much? How fast? How soon? Three simple concepts for progressing training loads to minimize injury risk and enhance performance. Journal of orthopaedic & sports physical therapy. 2020; 50(10): 570–573. 31726926 10.2519/jospt.2020.9256

[10] Holgado D, & Sanabria D. Does self-paced exercise depend on executive processing? A narrative review of the current evidence. International Review of Sport and Exercise Psychology. 2021; 14(1): 130–153.

[11] Lockie R G, Liu T M, Stage A A, Lazar A, Giuliano D V, Hurley J M, et al. J. Assessing repeated-sprint ability in Division I collegiate women soccer players. The Journal of Strength & Conditioning Research. 2020; 34(7): 2015–2023. 10.1519/JSC.0000000000002527 29702517

[12] Prasanth H, Caban M, Keller U, Courtine G, Ijspeert A, Vallery H, et al. Wearable sensor-based real-time gait detection: A systematic review. Sensors. 2021; 21(8): 2727. doi: 10.3390/s21082727 33924403 PMC8069962

[13] Blauberger P, Horsch A, & Lames M. Detection of ground contact times with inertial sensors in elite 100-m sprints under competitive field conditions. Sensors. 2021; 21(21): 7331. doi: 10.3390/s21217331 34770638 PMC8587724

[14] Saboor A, Kask T, Kuusik A, Alam M M, Le Moullec Y, Niazi I K, et al. Latest research trends in gait analysis using wearable sensors and machine learning: A systematic review. Ieee Access. 2020; 8: 167830–167864.

[15] Van Eetvelde H, Mendonça L D, Ley C, Seil R, & Tischer T. Machine learning methods in sport injury prediction and prevention: a systematic review. Journal of experimental orthopaedics. 2021; 8: 1–15. 33855647 10.1186/s40634-021-00346-x PMC8046881

[16] Al-Faris M, Chiverton J, Ndzi D, & Ahmed A I. A review on computer vision-based methods for human action recognition. Journal of imaging. 2020; 6(6): 46. doi: 10.3390/jimaging6060046 34460592 PMC8321068

[17] Su B, Smith C, & Gutierrez Farewik E. Gait phase recognition using deep convolutional neural network with inertial measurement units. Biosensors. 2020; 10(9): 109. doi: 10.3390/bios10090109 32867277 PMC7558451

[18] Zhang X, Beram S M, Haq M A, Wawale S G, Buttar A. M. Research on algorithms for control design of human–machine interface system using ML. International Journal of System Assurance Engineering and Management. 2022; 13(Suppl 1): 462–469.

[19] Serpush F, & Rezaei M. Complex human action recognition using a hierarchical feature reduction and deep learning-based method. SN Computer Science. 2021; 2(2): 94. doi: 10.1007/s42979-021-00484-0 33615240 PMC7881322

[20] Muhammad K, Ullah A, Imran A S, Sajjad M, Kiran M S, Sannino G, et al. Human action recognition using attention based LSTM network with dilated CNN features. Future Generation Computer Systems. 2021; 125: 820–830.

[21] Khan I U, Afzal S, & Lee J W. Human activity recognition via hybrid deep learning based model. Sensors. 2022; 22(1): 323. doi: 10.3390/s22010323 35009865 PMC8749555

[22] Zadeh S M, MacDermid J, Johnson J, Birmingham T B, & Shafiee E. Applications of wearable sensors in upper extremity MSK conditions: a scoping review. Journal of NeuroEngineering and Rehabilitation. 2023; 20(1): 158. doi: 10.1186/s12984-023-01274-w 37980497 PMC10656914

[23] Arunnehru J, Thalapathiraj S, Dhanasekar R, Vijayaraja L, Kannadasan R, Khan A A, et al. Machine vision-based human action recognition using spatio-temporal motion features (STMF) with difference intensity distance group pattern (DIDGP). Electronics. 2022; 11(15): 2363.

[24] Topic A, Russo M. Emotion recognition based on EEG feature maps through deep learning network. Engineering Science and Technology, an International Journal. 2021; 24(6): 1442–1454.

[25] Kanko R M, Laende E K, Strutzenberger G, Brown M, Selbie W S, DePaul V, et al. Assessment of spatiotemporal gait parameters using a deep learning algorithm-based markerless motion capture system. Journal of Biomechanics. 2021; 122: 110414. doi: 10.1016/j.jbiomech.2021.110414 33915475

[26] Pandy M G, Lai A K, Schache A G, & Lin Y C. How muscles maximize performance in accelerated sprinting. Scandinavian Journal of Medicine & Science in Sports. 2021; 31(10): 1882–1896. doi: 10.1111/sms.14021 34270824

[27] Liaqat S, Dashtipour K, Arshad K, Assaleh K, Ramzan N. A hybrid posture detection framework: Integrating machine learning and deep neural networks. IEEE Sensors Journal. 2021; 21(7): 9515–9522.

[28] Kalema R N, Schache A G, Williams M D, Heiderscheit B, Siqueira Trajano G, & Shield A J. Sprinting biomechanics and hamstring injuries: Is there a link? A literature review. Sports. 2021; 9(10): 141. doi: 10.3390/sports9100141 34678922 PMC8540816

[29] Abayomi-Alli O O, Damaševičius R, Misra S, Maskeliūnas R. Cassava disease recognition from low-quality images using enhanced data augmentation model and deep learning. Expert Systems. 2021; 38(7): e12746.

[30] Ma J, Jiang X, Fan A, Jiang J, Yan J. Image matching from handcrafted to deep features: A survey. International Journal of Computer Vision. 2021; 129(1): 23–79.

[31] Sun Y, Liu B, Yu X, Yu A, Gao K, Ding L. Perceiving spectral variation: Unsupervised spectrum motion feature learning for hyperspectral image classification. IEEE Transactions on Geoscience and Remote Sensing. 2022; 60: 1–17.

[32] Onyema E M, Shukla P K, Dalal S, Mathur M N, Zakariah M, Tiwari B. Enhancement of patient facial recognition through deep learning algorithm: ConvNet. Journal of Healthcare Engineering. 2021; 2021(1): 5196000. doi: 10.1155/2021/5196000 34912534 PMC8668299

[33] Mohan A, Singh A K, Kumar B, Dwivedi R. Review on remote sensing methods for landslide detection using machine and deep learning. Transactions on Emerging Telecozmmunications Technologies. 2021; 32(7): e3998.

[34] Al-Qurishi M, Khalid T, Souissi R. Deep learning for sign language recognition: Current techniques, benchmarks, and open issues. IEEE Access. 2021; 9: 126917–126951.

[35] Snoun A, Bouchrika T, Jemai O. Deep-learning-based human activity recognition for Alzheimer’s patients’ daily life activities assistance. Neural Computing and Applications. 2023; 35(2): 1777–1802.

